# Clinical challenges of tissue preparation for spatial transcriptome

**DOI:** 10.1002/ctm2.669

**Published:** 2022-01-26

**Authors:** Xiaoxia Liu, Yujia Jiang, Dongli Song, Linlin Zhang, Guang Xu, Rui Hou, Yong Zhang, Jian Chen, Yunfeng Cheng, Longqi Liu, Xun Xu, Gang Chen, Duojiao Wu, Tianxiang Chen, Ao Chen, Xiangdong Wang

**Affiliations:** ^1^ Department of Pulmonary and Critical Care Medicine Institute for Clinical Science Shanghai Institute of Clinical Bioinformatics Zhongshan Hospital of Fudan University Shanghai Engineering Research for AI Technology for Cardiopulmonary Diseases Shanghai China; ^2^ BGI Shenzhen China; ^3^ BGI College & Henan Institute of Medical and Pharmaceutical Sciences Zhengzhou University Zhengzhou China; ^4^ Jinshan Hospital Centre for Tumor Diagnosis and Therapy Fudan University Shanghai Medical College Shanghai China; ^5^ Institute of Computer Science Fudan University Shanghai China; ^6^ Shanghai Biotechnology Corporation Shanghai China; ^7^ Shanghai Lung Cancer Center Shanghai Chest Hospital Shanghai Jiao Tong University Shanghai China; ^8^ Department of Pathology Zhongshan Hospital, Fudan University Shanghai China; ^9^ Shanghai Lung Cancer Center Shanghai Chest Hospital Shanghai Jiao Tong University Shanghai China

**Keywords:** clinical challenge, critical factor, spatial transcriptomics, spatiotemporal molecular image, spatiotemporal molecular medicine

## Abstract

Spatial transcriptomics is considered as an important part of spatiotemporal molecular images to bridge molecular information with clinical images. Of those potentials and opportunities, the excellent quality of human sample preparation and handling will ensure the precise and reliable information generated from clinical spatial transcriptome. The present study aims at defining potential factors that might influence the quality of spatial transcriptomics in lung cancer, para‐cancer, or normal tissues, pathological images of sections and the RNA integrity before spatial transcriptome sequencing. We categorised potential influencing factors from clinical aspects, including patient selection, pathological definition, surgical types, sample harvest, temporary preservation conditions and solutions, frozen approaches, transport and storage conditions and duration. We emphasis on the relationship between the combination of histological scores with RNA integrity number (RIN) and the unique molecular identifier (UMI), which is determines the quality of of spatial transcriptomics; however, we did not find significantly relevance between them. Our results showed that isolated times and dry conditions of sample are critical for the UMI and the quality of spatial transcriptomic samples. Thus, clinical procedures of sample preparation should be furthermore optimised and standardised as new standards of operation performance for clinical spatial transcriptome. Our data suggested that the temporary preservation time and condition of samples at operation room should be within 30 min and in ‘dry’ status. The direct cryo‐preservation within OCT media for human lung sample is recommended. Thus, we believe that clinical spatial transcriptome will be a decisive approach and bridge in the development of spatiotemporal molecular images and provide new insights for understanding molecular mechanisms of diseases at multi‐orientations.

## Graphical abstract

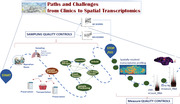

## 1 INTRODUCTION

With rapid development of biotechnologies, the understanding of medicine is extended from traditional medicine to molecular medicine. Recently, a new concept of spatiotemporal molecular medicine is coined proposed to understand ‘medicine’ at four dimensions of length, width, height and time, by integrating clinical spatialisation and temporalisation of phenomes with spatiotemporal molecular phenomes.^1^ Different from traditional medicine, medicine and molecular medicine, the spatiotemporal molecular medicine adopts the digital information of spatial and temporal transcriptomics, proteomics, metabolomics and other omics for the development of molecule‐targeted, location‐based and dynamics‐monitored diagnosis and therapy for patients. Spatiotemporal molecular medicine is an interdisciplinary subject between medicine, trans‐omics and bioinformatics. As one of important parts in spatiotemporal molecular medicine, the spatiotemporal molecular image information of spatiotemporal molecular is proposed as to show the dynamics and positioning of clinical molecular events in dynamics clinical images, for example, X‐ray, computerised tomography, electrocardiogram, nuclear magnetic resonance, positron emission tomography‐CT, ultrasound, tomography‐CT and interventional radiology and electrocardiogram.^2^ Those clinical images can be used to visualise a three‐dimensional architecture of organs by images information integration of multiple transverse and longitudinal organ sections of the organ images. Obtaining, comprehensive analyses, modelling and visualisation of multiple types of images of the same location at organ, histological types, cell, protein expression and phosphorylation and gene expression and epigenetic regulation is the key issue of spatiotemporal molecular atlas.

The development of spatiotemporal measurements and analyses is a limiting factor in the clinical applications of spatiotemporal molecular medicine. Of those, spatial transcriptomics is a new and critical way to define mRNA positioning and transcriptomic profiles on tissue section by measuring and visualising labelled arrayed reverse transcription primers with unique barcodes.^3^ Spatial transcriptomes describe new cell clusters, cell–cell interactions, transcriptional signals and phenotypes within the certain area of histological sections, through which clinical images are referred and corresponded. Spatial transcriptomics is considered as an important part of spatiotemporal molecular images to bring molecular information and clinical images together. Spatial transcriptome provides a potential to understand comprehensive profiles of cellular heterogeneity at a single cell resolution with a clear localisation corresponding to pathological changes. Clinical spatial transcriptome emphasises the integration of clinical phenomes with the comprehensive molecular understanding of pathological gene expression profiles and locations, cell–cell interactions, cell heterogeneity and transcriptomic changes of human tissues at single‐cell resolution. In addition to spatial transcriptome, spatiotemporal molecular pathology also includes spatial proteomes, metabolomes, lipidomes and pathological phenomes, although there are still many challenges to be faced and overcome.

There is a critical need to obtain a comprehensive snapshot of the preparation of spatial transcriptome for cellular heterogeneity at a single‐cell resolution, although there are still huge missing unmet needs to translate the technology into clinical practice.^4^ A number of transcriptomic profiling technologies based on in situ gene expression were developed, including laser capture microscopy coupled with full‐length mRNA‐sequencing (LCM‐seq), geographical position sequencing (GEO‐seq), multiplexed error‐robust FISH (MERFISH), sequential fluorescence in situ hybridisation (seqFISH), fluorescent in situ RNA sequencing (FISSEQ), spatially resolved transcript amplicon readout mapping (STARmap), Slide‐seq, spatial transcriptomics (ST, Visium) and their optimised versions Slide‐seqV2 high‐definition spatial transcriptome (HDST), deterministic barcoding in tissue for spatial omics sequencing (DBiT‐seq) and Stereo‐sEquation (Spatio‐Temporal Enhanced REsolution Omics‐sequencing) which we used currently. MERFISH and seqFISH are techniques based on pre‐designed probes and hybridisation‐based methods. Slide‐seq, Visium, HDST, DBiT‐seq and Stereo‐seq were using barcoded arrays. These methodologies using physical segmentation and pre‐designed targeted probes depend on RNA capture and tissue imaging.^5^ Thus, the RNA quality and imaging quality, detected by RNA integrity number (RIN) and haematoxylin and eosin (HE) staining, could be the key elements to evaluate the preparation of transcriptomic profiling. In addition to the sufficiency and precision of pathological region sizes and locations from biopsy tissues and the high cost of spatially resolved transcriptomics, one challenge in clinical application is the poor understanding on the value and significance of spatial transcriptomes, the preparations and analyses of human tissues and data and the comprehensive indications. Another challenge is to define proper conditions and operations of harvesting, handlings and preparations of deliveries and settle up an efficient monitoring of quality for spatial transcriptome, when human samples are preserved and processed during clinical practice.^6^, ^7^ The present study aims to explore potentials of clinical challenges to be faced during human tissue preparations for spatial transcriptome, including the selection, harvesting, preservation, transportation, processing, storage, quality control and sequencing from clinical operation room to spatial sequencing bench. This is one of the critical steps of spatiotemporal molecular images to translate the technology of spatial transcriptome to clinical practice, since the process of various human biopsies obtained from different surgical settings and handles is a decisive factor of the success of spatial transcriptomics for the clinical application.

## 2 METHODS

### 2.1 Patients and samples

About 77 clinical samples of lung cancer and patient‐matched normal and para‐cancerous lung samples were collected from 33 patients with lung cancer at the Zhongshan Hospital of Fudan University, Shanghai Chest Hospital and Shanghai Pulmonary Hospital. The lung cancer was classified according to the 2015 WHO classification,^8^ including non‐small cell lung cancer (NSCLC) and small cell lung cancer (SCLC), sarcomatoid carcinomas, as well as tumours with respective mixed components. The group of NSCLC was further subdivided into lung adenocarcinoma (LUAD), lung squamous cell carcinomas (LUSC) and large cell carcinomas. This study was approved by the Ethics Committee of Zhongshan Hospital of Fudan University (B2021‐265). The written informed consent was obtained from all patients or their guardians. All diagnoses were confirmed through histological examination.

### 2.2 Collection and transport of clinical specimens

**FIGURE 1:**
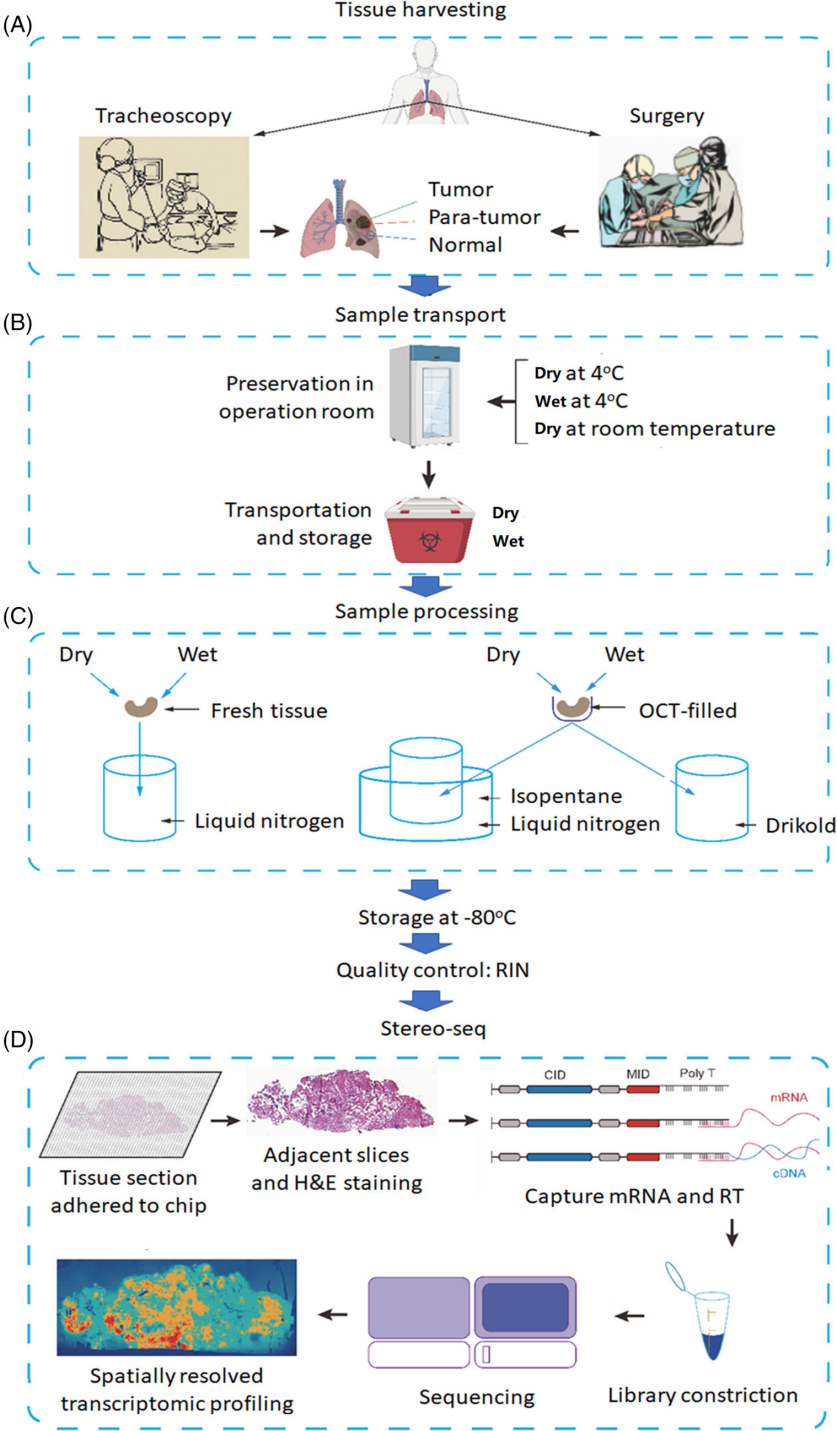
Processes and workflow of clinical spatial transcriptome from sample harvest to transcriptomic imprints. (A) Tissue harvesting: human samples of lung tumour, para‐tumour (>2 cm from cancer edge) and normal tissues (>3 cm from para‐cancer tissue) were harvested from either tracheoscopy or surgery. (B) Sample transport: the harvested tissues were temporarily maintained with physiological saline solution (‘wet’) or without solution (‘dry’) in the operating room at 4℃ or room temperature, then samples were transported to the laboratory from on ice. (C) Sample processing: tissues at wet or dry conditions were frozen directly in liquid nitrogen or were immersed with the solution of optimal cutting temperature compound (OCT) in isopentane cooled with liquid nitrogen or drikold. The processed samples were temporarily stored in the refrigerator at –80℃, and then RNA integrity number (RIN) value was detected. (D) Stereo‐seq: Spatio‐Temporal Enhanced Resolution Omics‐sequencing (Stereo‐Seq) is an emerging next generation of spatially resolved transcriptomics technology using the high resolution of regularly and septically spaced DNA nanobeads (DNB) to enable in situ RNA capture, amplification and sequencing

Processes and workflows of clinical spatial transcriptome from sample harvest at the operation room to transcriptomic imprints were described in Figure 1. Human samples of lung cancer tissues, para‐cancer tissues (>2 cm from cancer edge) and normal tissues (>3 cm from para‐cancer tissue) were harvested from either tracheoscopy or surgery at the operation rooms of the hospitals under various surgical settings with different methodologies (Figure 1A). Macroscopic characters of human lung tissue samples were imaged and recorded immediately from surgery and biopsies, after which the sample was divided for pathology and spatial transcriptome. The harvested tissues were put into the plate with physiological saline solution (called ‘wet’ here) or without solution (called ‘dry’ here) and temporarily maintained in the operating room at 4°C or room temperature for about 30 min (Figure 1B). The samples at wet or dry conditions were put into tubes on ice directly and transported from the operating room to the laboratory from 30 min to 2.5 h. The sample processing included unpacking, cryopreservation preparation, storage and quality control before sequencing, as shown in Figure 1C. Tissues at wet or dry conditions were frozen directly in liquid nitrogen or were immersed with the solution of optimal cutting temperature compound (OCT) in isopentane cooled with liquid nitrogen or drikold for 15 min followed by freezing in liquid nitrogen. The Stereo‐Seq is an emerging next generation of spatially resolved transcriptomics technology using the high resolution of regularly and septically spaced DNA nanobeads (DNB) to enable in situ RNA capture, amplification and sequencing.^5^ We adopted the standardised the Beijing Genome Institute (BGI) Stereo‐Seq sample processing protocol and performed sample quality controls and spatial sequencing. Total RNA was extracted from tissues with RNeasy Mini Kit (QIAGEN, Germany), and the RIN were analysed with Agilent 2100 Bioanalyzer (Agilent Technologies, USA).

### 2.3 Tissue sectioning and processing

STOmics Gene Expression Set‐S1 kit (http://manual.stereomics.com/Stereo‐Draftsman/STOmics_Gene_Expression_kit_S1_manual_Chinese or http://manual.stereomics.com/Stereo‐Draftsman/STOmics_Gene_Expression_kit_S1_manual_English) was used for spatial transcriptomics experiment. The tissues were taken out of –80°C freezer and equilibrated in the freezing microtome for 30 min. The tissues for experiment were cryo‐sectioned at 10 μm thickness in a Leika CM1950 cyrostat (Leica Mikrosysteme Vertrieb GmbH, Wetzlar, Germany). The tissue sections for spatial transcriptomics experiment were attached to the probe surface of the Stereo‐seq chip. The chips were incubated at 37°C for 3 min and immersed in the methanol at –20°C for 30 min to fix. After drying, the chips were permeabilised in 37℃ for 12 min using 0.1% PR Enzyme (BGI, Shenzhen, China) at pH 2.0 for subsequent RNA capturing. The tissue sections for HE staining, which adjacent to the spatial transcriptomics experiment sections, were immersed in the methanol at –20℃ for 30 min and stained with haematoxylin and eosin (Solarbio, Beijing, China), then imaged with a Motic microscope (Motic China Group, Xiamen, China) and could be download at https://pan.baidu.com/s/1l1nSxd2vwYnn0Ty5Bi5e_w. The quality scores of HE staining sections were obtained by two pathologists, respectively, and the average value of quality scores was taken for further calculation.

### 2.4 In situ reverse transcription and amplification (Figure 1D)

After permeabilisation, the chips were washed twice with 0.1 × SSC Buffer (Thermo, CA, USA) with 5% RI (BGI, Shenzhen, China). The chips were reversely transcribed for 1.5 h at 42℃ using RT Mix (80 μl RT Reagent, 5 μL RT Additive, 5 μl RI, 5 μl RT Oligo, 5 μl Reverse T Enzyme, a total of 100 μl mix per chip, BGI, Shenzhen, China). After the reverse transcription, the chips were washed twice with 0.1× SSC Buffer (Thermo, CA, USA) and digested with the TR Buffer (BGI, Shenzhen, China) at 37℃ for 30 min to remove tissue on the surface of the chip. The chips were washed twice with 0.1× SSC Buffer (Thermo, CA, USA). And then the chips were immersed in the cDNA Release Mix (20 μl cDNA Release Enzyme, 380 μl cDNA Release Buffer, a total of 400 μl mix per chip, BGI, Shenzhen, China) at 55℃ for 3 h to release the cDNA on the surface of the chip. The mix after reaction were collected, and the chips were washed 10 times with nuclease‐free H_2_O (Thermo, CA, USA). The reaction mix and the cleaning nuclease‐free H_2_O were mixed, and the new cDNA mix was purified using the Ampure XP Beads (Vazyme, Nanjing, China) with 0.8 times the volume of the new mix. The cDNAs were eluted with nuclease‐free H_2_O, and the recovered volume was 42 μl. The resulting cDNAs were amplified with PCR Mix (42 μl Eluted cDNA, 50 μl cDNA Amplification Mix, 8 μl cDNA Primer, a total of 100 μl mix per reaction, BGI, Shenzhen, China). The PCR reactions were the heated lid at 105℃, the first program at 95℃ for 5 min, the second program at 98℃ for 20 s, 58℃ for 20 s, 72℃ for 3 min with 15 cycles and the third program at 72℃ for 5 min.

### 2.5 cDNA library construction and sequencing

The PCR amplification products were purified using the Ampure XP Beads (Vazyme, Nanjing, China) with 0.6 times the volume of the products. The purified cDNA concentrations were measured with Qubit™ dsDNA HS Kit (Thermo, CA, USA). A total of 20 ng of purified cDNA products were fragmented with Fragmentation Mix (4 μl TMB, 1 μl 10‐fold diluted TME, x μl cDNA product, 15‐x μl nuclease‐free water, a total of 20 μl mix per reaction, BGI, Shenzhen, China) at 55℃ for 10 min. After fragmentation, the mix was added 5 μl Stop Buffer to terminate fragmentation and incubated at room temperature for 5 min. The fragmentation products were amplified using PCR Mix (25 μl Fragmentation Mix, 50 μl PCR Amplification, 25 μl PCR Barcode Primer Mix, a total of 100 μl mix per reaction, BGI, Shenzhen, China). The PCR reactions were the heated lid at 105℃, the first program at 95℃ for 5 min, the second program at 98℃ for 20 s, 58℃ for 20 s, 72℃ for 30 s with 13 cycles and the third program at 72℃ for 5 min. The cDNA mix of PCR amplification products was purified using the Ampure XP Beads (Vazyme, Nanjing, China) with first 0.6 times the volume of the mix and then 0.2 times the volume of the mix. The purified cDNA mix was used for DNB generation and finally sequenced paired‐end 50 bp on MGISEQ200 and MGI DNBSEQ‐T1 sequencer (MGI, Shenzhen, China).

The cell type was annotated by the combination of single‐cell sequencing cell annotation software SingleR and marker genes expression. The cell data of human lung in SingleR were selected as the reference database, then the correlation between the predicted cells and the reference database was calculated. The type annotation of the predicted cells was obtained after circularly calculating the correlation by continuously eliminating the type with the worst correlation.

### 2.6 Stereo‐seq spatial transcriptome data analysis

After sequencing, fastq files were generated and uploaded to the Stereo‐seq visual system (http://stereomap.cngb.org/) and CNGB Sequence Archive (https://db.cngb.org/) to finally process to form matrix (https://202.108.211.75). The expression matrix was divided into bins, which non‐overlap and cover the area of 50 × 50 DNB spots. And data were normalised and unsupervised clustered using Seurat. Then, normalisation and identification of highly variable genes were performed by SCTransform. Next, the dimensional reduction was processed by the runPCA. Finally, two‐dimensional data projections were obtained by the runUMAP function with dims = 30, and all clusters within the dataset were identified by the FindClusters with resolution = 0.3. The in‐house script is available at http://stereomap.cngb.org/ and in Supplemental File 1.^5^

### 2.7 Statistical analysis

All statistical data were analysed using SPSS 22.0 (SPSS, Chicago, IL, USA) and GraphPad 7.0 (GraphPad Software, San Diego, CA, USA). According to the results of the data normality test, the paired *T* test or Wilcoxon matched‐pairs signed‐rank test was applied to compare differences of HE and RNA integrity number (RIN) score between the normal and tumour groups, and ANOVA or nonparametric tests were used to compare the paired normal, para‐tumour and tumour groups. The unpaired *T* test or nonparametric Mann–Whitney U test was used to compare the two sets of data. The associations between the pathological characteristics of patients and the different sample methods of collection and processing with the RIN and HE score were analysed using the chi‐squared test. A two‐tailed *p* value of less than .05 was considered statistically significant in all tests.

## 3 RESULTS

**FIGURE 2:**
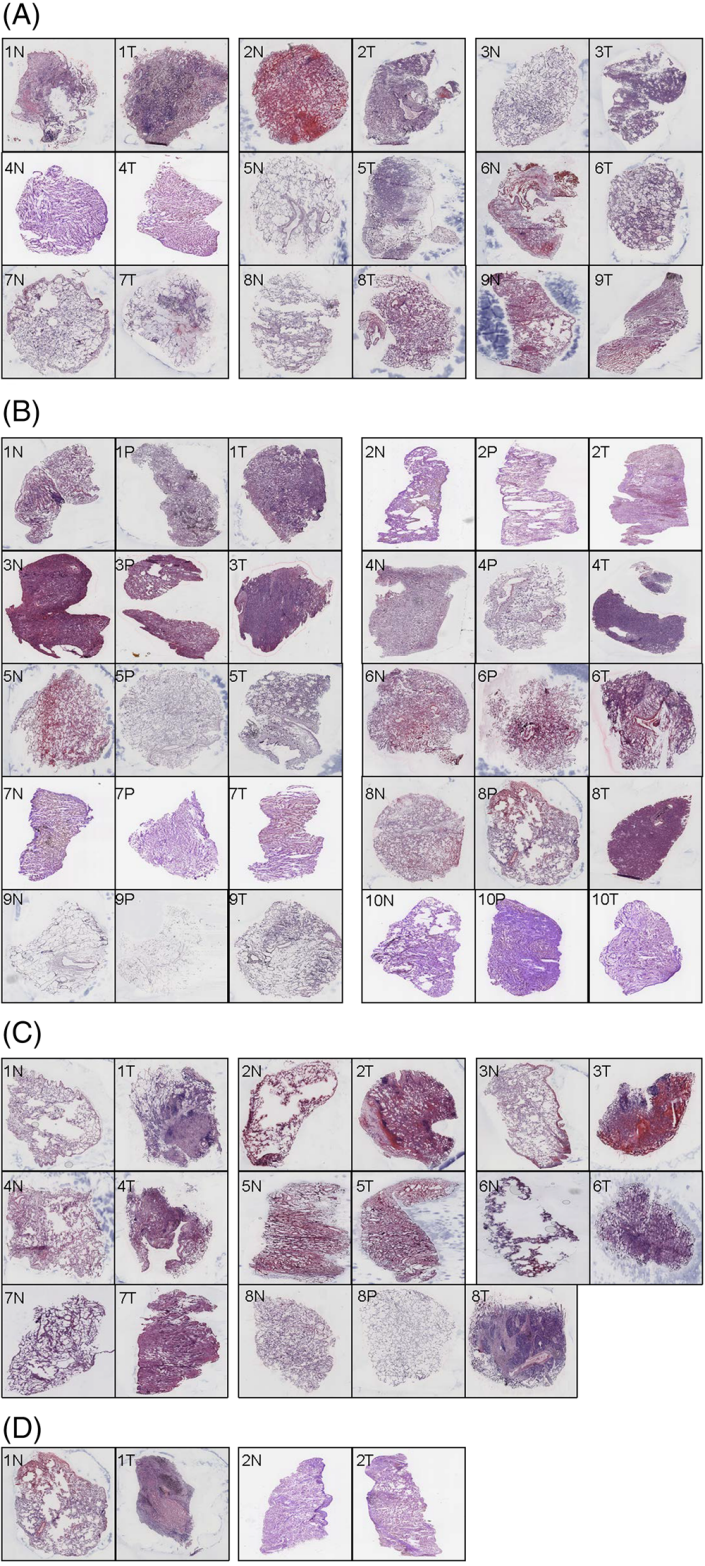
The haematoxylin and eosin (HE) staining images of different lung cancer types in paired samples, including normal tissues (N) and tumour tissues (T) or para‐tumour tissues(P). (A) Lung adenocarcinoma (LUAD: 9 N+T), 1N: the lung tissue structure was present, some alveoli collapsed and a few lymphocytes were infiltrated, 1T: the tumour cells showed invasive growth with less cytoplasm, hyperchromatic nuclei and diffuse infiltration, 2N: lung tissue structure was present and red blood cells were seen in alveolar cavities, 2T: adenocarcinoma cells were seen at the margins of the tissue, growing in an alveolar manner, 3N: normal lung tissue with minimal lymphocytic infiltration, 3T: LUAD with acinar growth, 4N: normal lung tissue with widened alveolar septum and mild fibrous hyperplasia, 4T: not clear enough to be recognised, 5N: the lung structure was basically normal, 5T: LUAD, mainly papillary type, a small amount of wall growth type, 6N: normal lung tissue with red blood cells in alveoli, 6T: LUAD, mainly with wall neoplasia, 7N: the lung structure was normal, 7T: mucinous adenocarcinoma with adherent growth, 8N: the lung tissue was basically normal, 8T: lung cancer mainly with adherent growth, 9N: normal lung tissue, some alveolar structures collapsed, 9T: not clear enough to be recognised. (B) LUAD: 10 N+P+T, 1N: normal lung tissue with minimal lymphocytic infiltration, 1P: the alveolar septum widened with mild fibrous hyperplasia and more dust deposition in lymphatic vessels, 1T: LUAD, adherent growth type, 2N: lung tissue was generally normal with mild fibrosis of the alveolar septum, 2P: alveolar interstitial fibrosis, no tumour tissue, 2T: small amount of acinar type tumour tissue, 3N: the alveolar structure collapsed with lymphocytic infiltration, 3P: alveolar interstitial fibrosis, 3T: LUAD, acinar type, 4N: widened alveolar septum with mild fibrosis, 4P: widened alveolar septum, 4T: LUAD, mainly acinar type, a few papillary type, 5N: lung tissue with some red blood cells, 5P: the lung structure was normal, 5T: a small number of tumour cells grew adherent to the wall, 6N: small amount of lung tissue with red blood cells, 6P: small amount of lung tissue with alveolar septum fibrous hyperplasia, 6T: LUAD, acinar type with marginal adherent growth, 7N: lung septum widened with fibrous tissue mildly hyperplasia, 7P: similar to 7N, 7T: a small number of heterotypic cells, adherant growth, 8N: normal, 8P: the alveolar septum widened with mild fibrous hyperplasia, 8T: LUAD, mainly acinar, some solid, 9N: normal, 9P: the organisational structure was incomplete, 9T: LUAD with adherent growth, 10N: normal, 10P: LUAD, mainly adherent growth, 10T: a small number of tumour cells grew adherent to the wall and mucus was visible in the alveolar cavity. (C) Lung squamous cell carcinomas (LUSC: 7 N+T & 1 N+P+T), 1N: normal, 1T: a large amount of mucus in the alveolar cavity and tumour cells grew adherent to the wall, 2N: basically normal, 2T: LUAD, signet ring cell type, 3N: normal lung tissue with red blood cells, 3T: the alveolar cavity was dilated, red blood cells were found in the cavity and a few lymphocytes were infiltrated in the stroma, none obvious tumour cells were found, 4N: normal, 4T: poorly differentiated carcinoma with solid growth, 5N: normal lung tissue with red blood cells in alveolar cavities, 5T: a small number of cancer cells grew in sheets, and red blood cells were seen in alveolar cavities, 6N: structure is incomplete, 6T: atypia cells, 7N: normal, 7T: small nodular lesions were observed without obvious cancer cells; 8N: basically normal, 8P: normal, 8T: poorly differentiated squamous cell carcinoma, nonkeratinised type. (D) Non‐small cell lung cancer (NSCLC: 2 N+T), 1N: normal, 1T: large necrosis was observed in the lesion, with more dust deposition, and no obvious malignant cells were observed, 2N: a small amount of fibrous hyperplasia in the alveolar septum, 2T: small nodular lesions were observed in the tissue with fibrous hyperplasia, none obvious malignant cells were observed

There were 125 samples from 57 pairs of lung cancer, para‐cancer or normal tissues, of which 77 samples of 33 pair tissues were harvested in the discovery study and 48 samples of 24 pair tissues in the validation study. Of 125 samples, 54 samples were qualified for spatial transcriptomes, with 43% successful rate of preparation. Clinical phenomes of patients were summarised in Table 1, including 67% male, 70% age ≥60, 94% no family or previous history, 82% no‐smoking, 64% LUAD, 60% no adjuvant treatment, about 50% in T1 and no lymphatic metastasis, 90% M0 metastasis and about 50% in stage I of tumour‐node‐metastasis (TNM). To investigate the relationship between pathological features and spatial transcriptomics success, we summarised pathological features of 33 lung cancer cases and categorised the RIN into three groups (<6, 6–7 or ≥7) and the HE scores into two groups (<90 or ≥90), as shown in Table 2. We found that those clinical phenomes had no significant correlation with RIN or HE scores. To determine whether the sample quality was suitable for spatial transcriptome sequencing, the parameters of HE and RIN scores are listed in Table S1. Figure 2 showed the HE staining image and demonstrated pathological phenomes and numbers of samples, respectively, including 9 pairs of normal and LUAD tissues and 10 pairs of normal, para‐tumour and LUAD tissues (Figure 2A and B); 7 pairs of normal and LUSC tissues and 1 pair of normal, para‐tumour and LUSC tissues (Figure 2C) and 2 pairs of normal and NSCLC tissues (Figure 2D).

**TABLE 1:** The pathological features of lung cancer cases

Characteristics	Number of cases (%)	Characteristics	Number of cases (%)
**Gender**		**T classification**	
Female	11 (33.3)	**/**	3 (9.1)
Male	22 (66.7)	T1	14 (42.4)
**Age (years)**		T2	9 (27.3)
<60	10 (30.3)	T3	3 (9.1)
≥60	23 (69.7)	T4	4 (12.1)
**Family history of cancer**		**Lymphatic metastasis**	
No	31 (93.9)	**/**	3 (9.1)
Yes	2 (6.1)	Negative	19 (57.6)
**Previous cancer history**		Positive	11 (33.3)
No	31 (93.9)	**Metastasis**	
Yes	2 (6.1)	**/**	3 (9.1)
**Smoking history**		M0	30 (90.9)
No	27 (81.8)	M1	0 (0)
Yes	6 (18.2)	**TNM stage**	
**Pathological type**		**/**	1 (3.0)
LUAD	21 (63.6)	I	16 (48.5)
LUSC	8 (24.2)	II	3 (9.1)
NSCLC	4 (12.1)	III	11 (33.3)
**Adjuvant treatment**		IV	2 (6.1)
No	20 (60.6)		
Yes	13 (39.4)		

LUAD: lung adenocarcinoma, LUSC: lung squamous cell carcinomas, NSCLC: non‐small cell lung cancer, TNM: tumour‐node‐metastasis.

**TABLE 2:** The relationship between the pathological characteristics of patients with the RNA integrity number (RIN) and haematoxylin and eosin (HE) staining score

**Characteristics**	**RIN**				**HE score**		
	**<6**	**6**–**7**	**≥7**	***p* Value**	**<90**	**≥90**	***p* Value**
**Gender**							
Female	3	2	4	.88	2	7	1.00
Male	4	6	7		4	15	
**Age (years)**							
<60	1	1	4	.51	1	6	1.00
≥60	6	7	7		5	16	
**Smoking history**							
No	5	6	10	.56	3	19	.09
Yes	2	2	1		3	3	
**Pathological type**							
LUAD	5	5	7	.65	5	13	.77
LUSC	1	2	4		1	7	
NSCLC	1	1	0		0	2	
**Adjuvant treatment**							
No	2	5	8	.25	4	12	.67
Yes	5	3	3		2	10	
**T classification**							
T1	3	3	7	.61	4	9	.37
T2 & T3 & T4	4	5	4		2	13	
**Lymphatic metastasis**							
Negative	3	6	8	.45	4	14	1.00
Positive	4	2	3		2	8	
**TNM stage**							
I	2	5	7	.37	4	11	.66
II & III & IV	5	3	4		2	11	

LUAD: lung adenocarcinoma, LUSC: lung squamous cell carcinomas, NSCLC: non‐small cell lung cancer, TNM: tumour‐node‐metastasis.

**FIGURE 3:**
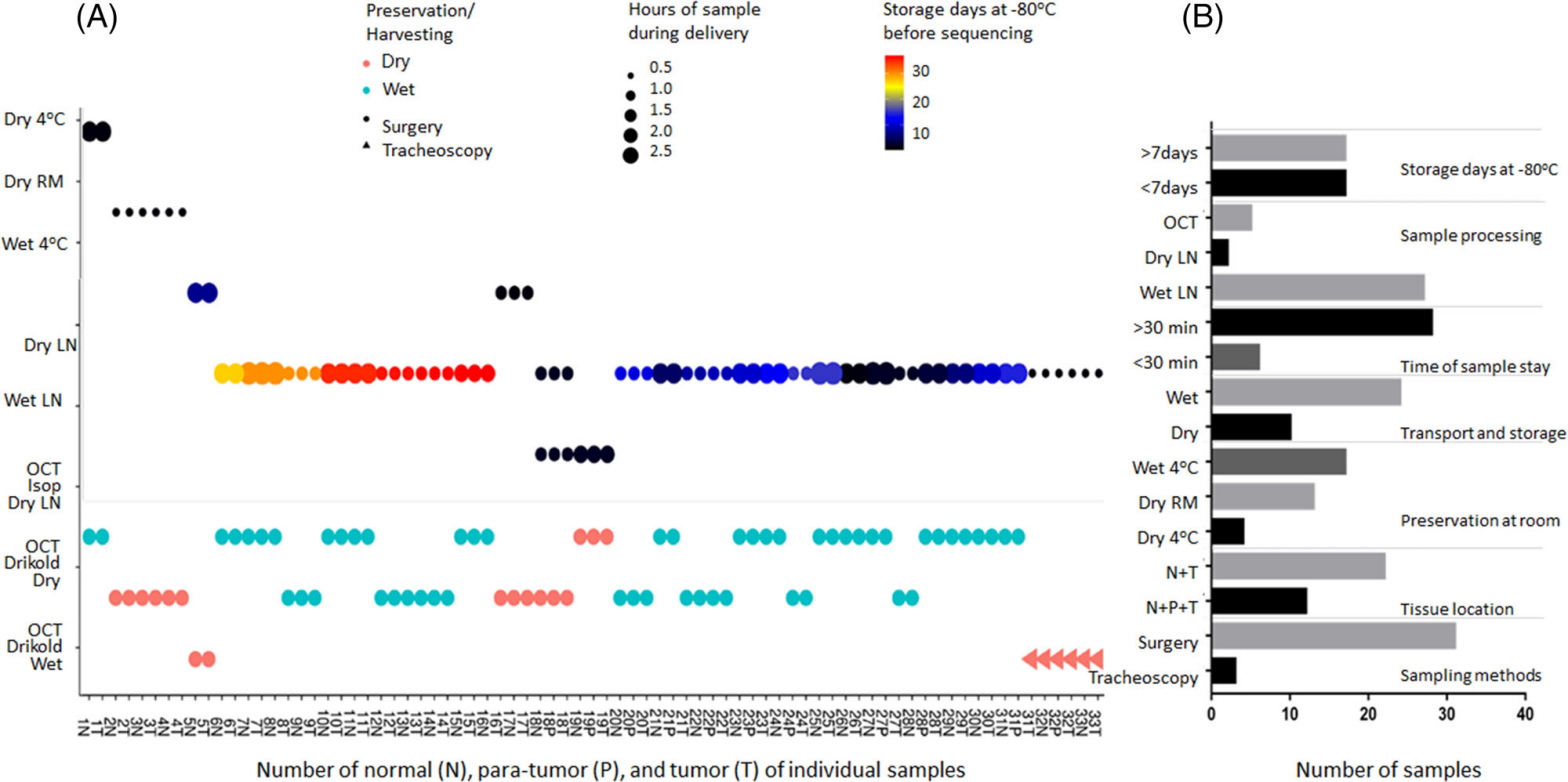
The collected and processed information for each sample. (A) Details of each tissue handling: the horizontal axis shows the patient number and indicates the tissue locations, the vertical axis shows different preservation methods at operation room and different sample processing methods, red and green, respectively, represent dry and wet condition in transport and storage, dots and triangles represent different sampling method, the size of the point was different delivery hours of samples and different colours express different storage days at –80℃. (B) The sample number of different processed method

**FIGURE 4:**
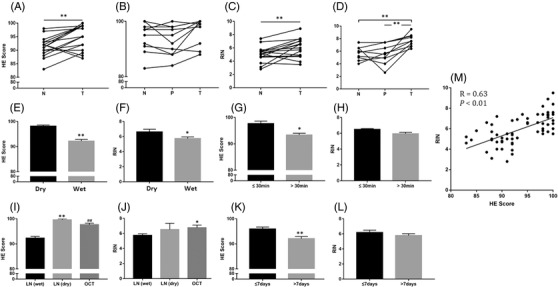
Influencing factors of HE and RIN score in sample collection and processing. Influencing factors of spatial transcriptome, including tissue location (A, B: HE score, C, D: RIN), transportation and storage (E: HE score, F: RIN), delivery hours of samples (G: HE score, H: RIN), sample processing method (I: HE score, J: RIN) and storage days at –80℃ (K: HE score, L: RIN). (M) The correlation between HE and RIN score

Influencing factors of samples were monitored and calculated for the quality of spatial transcriptome, according to sample collection and processing (Figure 1), including tissue location, sampling method, preservation in operation room, transportation and storage, delivery hours of samples, sample processing method and storage days at –80°C. Details of each tissue handling during preservation in the operation room and sample processing were shown in Figure 3. The information collected and processed for each sample was summarised in Table S2. We found the significant difference in HE scores between normal and tumour tissues (Figure 4A) and in RIN scores between normal and tumour tissues (Figure 4C) and between para‐tumour and tumour tissues (Figure 4D), respectively (*p *< .01 or less), rather than HE scores between para‐tumour and tumour tissues (Figure 4B). Levels of HE and RIN scores in wet tissues were significantly lower than those in dry (Figure 4E and F, *p *< .01 and .05, respectively). The HE scores of tissues in >30 min delivery were lower than those in ≤30 min (Figure 4G), while not in RIN scores (Figure 4H). HE and RIN scores of dry tissues in liquid nitrogen and in OCT‐immersed tissues were higher than those of wet tissues in liquid nitrogen (Figure 4I and J). HE scores in tissue stored at –80°C for >7 days were lower than ≤7 days (Figure 4K, *p *< .01) while not RIN score (Figure 4L). The correlation coefficient between HE and RIN scores was 0.63 with significance (Figure 4M, *p *< .01).

The values of influencing factors within RIN or HE scores were furthermore evaluated by the incidence of tissue samples in individual influencing factors (Table 3). About 60% of normal tissues and 55% of wet tissues had RIN scores <6, different from para‐tumour or tumour tissues and from dry tissue (*p *< .01). About 50% samples were preserved during 0.5–1.0 h and 1.5–2.0 h at operation room (*p <* .05) and stored as wet tissue in liquid nitrogen with RIN scores <6 (*p <* .05). The majority of tissues with storage days <7 at –80°C had higher HE scores (*p <* .05). All of dry tissues in liquid nitrogen and OCT‐immersed tissues had higher HE scores.

**TABLE 3:** The number of normal (N), para‐tumour (P) and tumour tissues (T) in influencing factors in groups categorised with RIN or HE scores

		**RIN scores**				**HE scores**		
**Influence factors**	**Total *n* = 80**	**< 6 *n* = 33**	**6–7 *n* = 14**	**≥7 *n* = 18**	***p* Value**	**< 90 *n* = 16**	**≥90 *n* = 53**	***p* Value**
**Tissue locations**								
N	34	20	5	3	**<.01**	7	22	.93
P	12	6	1	3		3	8	
T	34	7	8	12		6	23	
**Sampling methods**								
Tracheoscopy	6	/	/	/	/	/	/	/
Surgery	74	33	14	18		16	53	
**Preservation conditions in operation room**								
Dry	43	15	8	10	.73	8	29	1.00
Wet	37	18	6	8		8	24	
**Transportation and storage**								
Dry	26	3	7	6	**<.01**	0	19	**<.01**
Wet	54	30	7	12		16	34	
**Delivery hours of samples**								
≤0.5 h	12	0	2	0	**.02**	0	6	.14
0.5—1.0 h	29	14	5	10		8	21	
1.0—1.5 h	6	3	3	0		3	2	
1.5—2.0 h	26	15	4	4		5	19	
>2.0 h	7	1	0	4		0	5	
**Sample processing method**								
Liquid nitrogen (wet)	61	30	8	13	**.04**	16	35	**.02**
Liquid nitrogen (dry)	6	1	2	3		0	5	
OCT	13	2	4	3		0	13	
**Storage days at ‐80^o^C**								
≤7 days	40	11	8	9	.26	3	28	**.02**
>7 days	40	22	6	9		13	25	

OCT: optimal cutting temperature compound.

**FIGURE 5:**
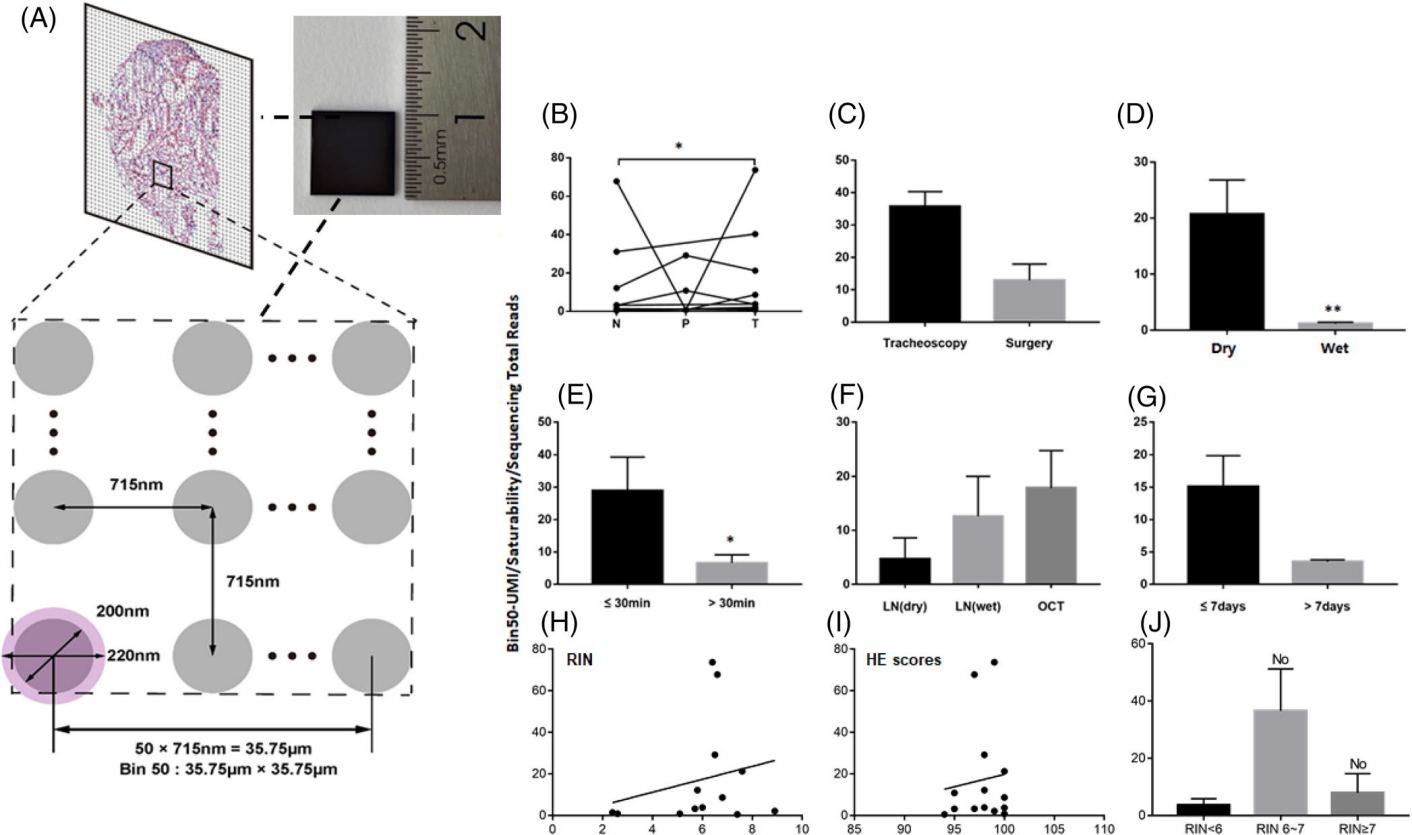
Quality control of the spatial transcriptome. (A) Image of Bin 50. Stereo‐seq chip surface arranges the spot patterned array chip. Chips have spots with 200 nm diameter and a centre‐to‐centre distance of 715 nm. Each DNB with a diameter of approximately 220 nm occupies a single spot. Probe on DNB captures mRNA from 10 μm thickness tissue sections covering the chip. The matrix region composed of 50 DNB spacing was named Bin50 for subsequent analysis. (B–G) Influencing factors of RNA capture efficiency, including tissue location (B), sampling method (C), transportation and storage (D), delivery hours of samples (E), sample processing method (F) and storage days at –80°C (G), RIN (H), HE scores (I), RIN < 6, 6–7 or > 7 (J). The Bin50‐UMI/saturability/sequencing total reads represent the RNA capture efficiency on basis of the amount of Bin50 capture, saturation and sequencing

We examined the quality of Stereo‐Seq data obtained in relation to each of variables (Table S3) to control the quality of the spatial transcriptome. From data allocation and analysis, we obtained an approximate measurement number of mRNA molecules successfully captured on a defined spatial resolution under identical sequencing depth by the Bin50‐UMI/saturability/sequencing total reads (BUSSTR). Bin50 is a matrix area where 50 capture points are combined together, unique molecular identifier (UMI) is an index to quantify the capture amount and Bin50‐UMI is to quantify the capture amount at the Bin50 level. The regional capture amount of Bin50 each slice was calculated and compared after the slice‐sequencing amount was normalised. DNBs were efficiently docked in each spot, approximately 220 nm in diameter and with a centre‐to‐centre distance of 715 nm. The Bin50 range diameter is the distance of 50 spot points, which is ∼35 μm (Figure 5A). The BUSSTR represents the RNA capture efficiency on basis of the amount of Bin50 capture, saturation and sequencing. Levels of tumour sample BUSSTR were significantly higher than those of the normal (Figure 5B, *p *< .05), dry sample BUSSTR higher than the wet (Figure 5D, *p *< .01) and sample BUSSTR of isolation time ≤30 min higher than the >30 min (Figure 5E, *p *< .05). There were no statistical differences between methods of tracheoscopy and surgery (Figure 5C) and preservation days at –80°C for ≤7 and >7 days (Figure 5G) or among storage conditions of dry and wet tissues in liquid nitrogen and OCT‐ immersed (Figure 5F). There were no statistical differences between RIN or HE scores and UMI (Figure 5 H and I). RIN < 6, RIN = 6–7 or RIN > 7 was analysed, which did not show statistical differences with UMI (Figure 5J).

**FIGURE 6:**
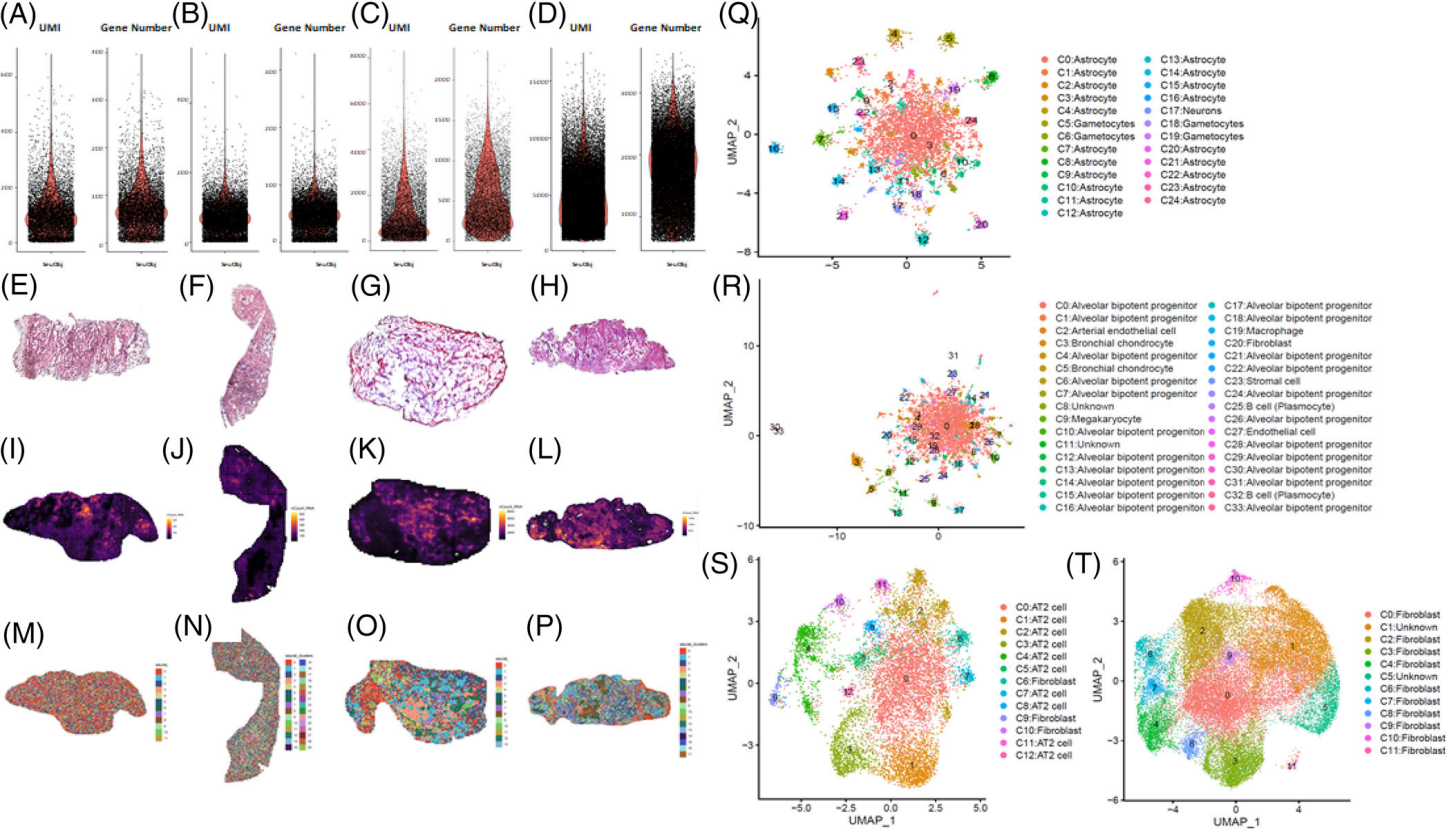
The visualised image of spatial transcriptomics quality control. (A–D) Violin plot showing the UMI (left) and gene number (right) captured by Stereo‐seq at Bin50 with different sample tissues of squamous cell lung cancer. A (normal) and B (tumour) from one patient, C (normal) and D (tumour) from another patient; (E‐H) HE staining of samples corresponding to the spatial transcriptomics results. (I‐L) Spatial visualisation of the number of UMI captured by Stereo‐seq from two samples at bin 50 resolution. E and I (normal) and F and J (tumour) from one sample, G and K (normal) and H and L (tumour) from another sample; (M‐P) Unsupervised spatially‐constrained clustering of the different sample sections analysed by Stereo‐seq data at bin 50 resolution. I (normal) and J (tumour) from one sample, K (normal) and L (tumour) from another sample. (Q‐T) UMAP projection of Stereo‐seq data at Bin 50 with clusters from four sections of two samples. Q (normal) and R(tumour) from one sample, S (normal) and T (tumour) from another sample. AT2: Type II alveolar epithelial cells

The visualised image of spatial transcriptomics for quality controls of two pairs (normal and tumour) samples were shown in Figure 6. Violin plots (Figure 6A–D) showed the number of UMI and genes captured by spatial transcriptomics within the range of Bin50. The mean number of UMI (left) and gene number (right) captured in the normal tissue section (Figure 6A) and the tumour tissue section (Figure 6B) of the first patient were less than 200 and 100, respectively. In the second patient, the mean number of UMI (Figure 6C, left) and gene number (Figure 6C, right) captured in the normal tissue section were less than 1000 and 500, while in the tumour, tissue sections were less than 3000 (Figure 6D, left) and 2000 (Figure 6D, right), respectively. HE staining of lung tissues corresponding to the spatial transcriptomics results is shown in Figure 6E–H. Spatial visualisations were obtained by spatially displaying the UMI captured in sample slices (Figure 6I–L). The overall darker colour of the normal and tumour tissue images in the first patient indicates low capture. The overall colours of normal and tumour tissue were brighter and were accompanied by higher capture numbers in the second patient. Different cluster distributions were obtained by unsupervised spatially constrained clustering of sample slice data (Figure 6M–P). The cluster distribution of the normal and tumour tissues of the first patient were chaotic (Figure 6M and N, respectively), which could hardly reflect the spatial local aggregation. The tissues of the second patient showed significantly different between the spatial local aggregation and distribution of tissue structures or cells (Figure 6O and P), which better reflected the true pathological status of the samples. Figure 6M–P showed the clustering results of sample slices using uniform manifold approximation and projection (UMAP). The tissue clustering in the first patient was poorly separated (Figure 6Q and R), with many scattered clusters, indicating that the cluster dimension reduced and clustering quality was poor. The high quality of clustering results of the two slices and separations of each cluster was noticed in the second patient, with better dimension reduction and clustering (Figure 6S and T). After clustering normal epithelial cells, 24 and 33 subpopulations were observed in normal tissue section (Figure 6Q) and the tumour tissue section (Figure 6R) of the first patient, respectively. We defined cell subsets by the expressing known representative genes in the normal tissue section, and astrocyte, neurons and gametocytes were defined (Figure 6Q), while the clusters defined in the tumour tissue section of the first patient mainly including alveolar bipotent progenitor. There were other clusters defined such as arterial endothelial, bronchial chondrocyte, megakaryocyte, macrophage, fibroblast, stromal cells, etc. (Figure 6R). Twelve and eleven subpopulations were observed in normal tissue section (Figure 6S) and the tumour tissue section (Figure 6T) of the second patient, respectively. The clusters mainly included type II alveolar epithelial cells and a few fibroblast clusters in the normal tissue section, and fibroblast clusters in the tumour tissue section (Figure 6T).

## 4 DISCUSSION

Spatial transcriptomics are considered as an important part of spatiotemporal molecular medicine and a critical bridge between spatial pathology and spatial molecular image.^1^, ^2^ The integration of gene positioning and distribution visualised indirectly from single‐cell RNA sequencing (scRNA‐seq) as well as gene location and profile observed directly from spatial transcriptomics will provide new insights of biological and pathological molecular mechanism.^9^ Spatial transcriptomes will be a new source to identify new pathology‐specific biomarkers and therapeutic targets, a revolutionary system to develop digital molecular pathology and dynamic monitor system, and an understanding of disease development to categorise disease subtypes and cell subclusters.^9^, ^10^, ^11^ Spatial transcriptomic profiles demonstrate cell–cell interactions in tissues, spatial relations between cell locations and transcriptional phenomes and evolutions of lineages and populations. According to the RNA capture approach, the spatial transcriptomic profiling techniques using DNA‐barcoded probes include Slide‐seq, Visium and HDST. The nature of captured transcripts was unbiased; however, there is unbalance between resolution, average numbers of gene per bin and capture rate between different bins. The measured areas of Visium, Slide‐seq/Slide‐seqV2, HDST and DBiT‐seq are 42.5, 7, 13.68 and 25 mm^2^, respectively.^3^, ^12^, ^13^, ^14^ Increasing the resolution and enlarging the capture area are important in the progress of transcriptomic profiling technique. Stereo‐seq is based on barcoded probes as well, but with a significantly larger spatial barcode pool size (425) and maintains sequence fidelity compared with bead‐based approaches by using rolling circle amplification in random barcode‐labelled DNB. The diameter of our capture spot is 200 nm, and the distance between points is 500/715 nm. Therefore, the transcriptomics resolution of Stereo‐seq is 500/715 nm, bringing about much higher number of spots per average size.^5^ Fu et al. described a technology for spatial transcriptome called polony (or DNA cluster)‐indexed library‐sequencing (PIXEL‐seq), which improved upon other spatial barcoding methods. This method could be with ≤1 μm resolution and captures >1000 unique molecular identifiers/10 × 10 μm^2^ by using ‘continuous’ polony oligos arrayed across a customised gel surface.^15^ Imaging‐based methods allowed for multiplex detection of a panel of proteins. hsrChST‐seq is indeed probably the first spatial epigenome sequencing study.^16^ The study performed spatial epigenome profiling for three histone modifications (H3K27me3, H3K4me3, H3K27ac) via hsrChST‐seq. Spatial chromatin state profiling in tissue may enable unprecedented opportunities to study epigenetic regulation, cell function and fate decision in physiology and pathogenesis situation.^17^ Rong Fan et al. reported spatially resolved chromatin accessibility profiling of mouse embryos delineated tissue region‐specific epigenetic landscapes and identified gene regulators implicated in the central nerve system development.^18^ The present study investigated potential factors that might influence the quality of spatial transcriptomics in lung cancer, para‐cancer or normal tissues and evaluated the quality of pathological images of sections by the quality HE staining for spatial transcriptomics and the RNA integrity determined by RIN values before spatial transcriptome sequencing. The RIN was recommended as one of optimal approaches to define the quality of the RNA integrity and as which is the first step in assessment of gene expression.^19^ However, our results did not show the significant relationship between histological scores >90 or RIN with and the UMI, which may mean that histological scores >90 will not affect the quality of spatial transcriptomic samples, while as small number of samples, RIN did not show the relationship with UMI as well. Thus, we tried to categorise potential challenges during tissue location, transportation and storage, delivery hours of samples, sample processing method, storage days at –80°C and responses from participates. Our results showed that isolated times and dry conditions of sample are critical for the UMI and the quality of spatial transcriptomic samples and suggested that the temporary preservation time and condition of samples at operation room should be within 30 min and in ‘dry’ status. The selection of samples for spatial transcriptome is highly dependent upon clinician and patient understanding, disease nature and severity and pathological size and location.

There is an increasing number and evidence to investigate spatiotemporal position of molecular events in patient samples, which are harvested from surgery, biopsies and post‐mortem. Due to the limit of human sample availability, the strategies of spatial transcriptomic studies include the reconstruction visualised by transcriptomic profiles of separated single cells harvested from the tissue, spatiotemporal dynamics of molecular pathology by direct tissue sequencing, combination of scRNA‐seq with tissue sequencing or integration of spatial transcriptome dynamics in animal models with target validation in human samples.^9^, ^20^, ^21^ For example, the spatiotemporal dynamics of the intact spinal tissue can be hardly performed in human, while it was carried out in mouse model of amyotrophic lateral sclerosis to define interactions between motor neurons and glia by screening dynamics and altars of spine and brain spatial transcriptomics.^20^ The pathway dynamics identified from the animal model were furthermore validated in human post‐mortem spinal cords, to confirm the similarity and difference between. Our preliminary data indicate that the precision selection and separation of human tissue specimens should be clearly defined for objectives of spatial research, including sample size, location, timing, handling and preserving in clinical practice. The tissue obtained by bronchoscopy in the present study was too small to be successful. It would be more valuable if pathological phenomes can be digitalised and collected before the sample delivery.

The histopathologic diagnosis and local lesion confirmation are also quality guarantees to ensure the precise information generated from spatial transcriptomics. The disease and patient selection, tissue acquisition and ethics are critical steps to perform spatial transcriptome studies in clinical samples. The questions were how the spatial transcriptomic profiles can help clinical diagnosis and therapy, what patients can be benefited from spatial positioning molecular information and how the quality of samples can be ensured to meet the criteria of spatial transcriptome. Answers to those questions are important for clinicians to make correct decisions of patient selection, for patients to offer the informed consent and for pathologists to define the special and precise location of specimens. The ethic permissions of human clinical trials and/or research for spatial transcriptomes require the guidelines, standardisation and procedures of sample resources, harvesting and handling and informed consent to participants, as demonstrated previously.^22^ Our study provides solid evidences of clinical sample handling, details of clinical practice and list of potential influencing factors for the maintenance of spatial transcriptome quality, future decision of sample standardisation and application of clinical ethics.

More and more studies on spatial transcriptome are undergoing human samples and clinical research/trial to deeply understand molecular mechanisms of human diseases and validate human disease‐specific biomarkers and targets. Spatial transcriptome‐driven biomarkers are expected like others to be disease severity‐, duration‐, stage‐, phenome‐ and therapy‐specific with the value of clinical application and impact of prognostic improvement.^23^, ^24^, ^25^, ^26^, ^27^

The sample preservation time at operation room and storage times at frozen were indirect factors, while the transportation methods from operation room to laboratories were direct factors to influence the quality of spatial transcriptome. After overviewing spatial transcriptomic studies on basis of human samples, those details were hardly noticed, due to the lack of sample handling reports, the focus on findings from spatial molecular positioning and the negligence of sample handling practice. Study on spatial transcriptomes of Stanford type A aortic dissection tissue section was focused on visualisation and analysis of gene expression and distribution of cell types and demonstrated that the sample preservation time was less than 30 min.^28^ Moncada et al. combined the microarray‐based spatial transcriptomics with scRNA‐Seq and visualised the distributions of various cell types with tissue regions.^21^ In this particular study, the preservation time of pancreatic ductal adenocarcinoma was about 2 h, although the successful rate of such samples was unclear. In clinical practice, the preservation time 30 min was at least selected and varied among surgeries, during which surgeons still needed to finish the lymph node dissection and the closure after the sample was harvested. It is critical to have standardised preservation/retention condition at operation room, in order to avoid the exposure of samples in room temperature or the delay due to personnel shorthand. Our data showed that the quality of tissue morphology was lower at the sample preservation time at operation room >30 min and storage times at frozen >7 days. The influence in in situ hybridisation with RNA probe needs to be furthermore validated in a large scale of populations. It is hard to identify the location and distribution of positive staining in the low quality of tissue morphology.

The variations of temporary preservation conditions and solutions directly influence the quality of tissue morphology and spatial transcriptome. Some studies on spatial transcriptome demonstrated that the tissue specimen was rinsed with cooled saline or in cooled PBS, for example, aortic dissection tissue,^28^ pancreatic ductal adenocarcinomas^21^ or human intestine,^29^ although there were no comparative published data to show potential effects of temporary preservation conditions. We compared ‘wet’ or ‘dry’ temporary conditions in the operating room at 4°C or room temperature for about 30 min. Our data showed that the lung tissues in ‘wet’ condition had lower levels of HE scores and RIN values and that the quality of lung samples embedded with OCT frozen in drikold was better than that in liquid nitrogen or drikold alone. A number of frozen conditions and solutions were used in the preparation of spatial transcriptome samples, including liquid nitrogen alone, liquid nitrogen‐precooled isopentane, liquid nitrogen‐precooled isopentane with OCT or frozen OCT.^21^, ^28^, ^29^, ^30^, ^31^, ^32^, ^33^ We selected the combination of drikold and OCT for the preparation of lung tissues, since the isopentane has limited access due to the toxicity and OCT has supportive effects in alveolar spaces. Such supportive effects of OCT may be invisible in the tumour with solid issues, since there was no difference between ‘dry’ and ‘dry’ lymph nodes with OCT. Our data suggest that the preservation time at –80°C should be maintained within 7 days before spatial sequencing. Gene capacities of sensitivity and resistance to preservation, storage and handling vary among individual gens per se, although the storage temperature and time of frozen sections could influence the RNA integrity and sequencing quality.^34^

The combination of tissue histological quality with RIN is a predictive factor of spatial transcriptome. The spatial RIN for rRNA completeness was suggested as an important criterium to define the quality of tissues for spatial transcriptome.^35^ We found that a few samples had the proper values of RIN, for example, around 7, while morphological images of lung alveoli and alveolar walls were blurred and indistinct, angulating or disintegrated. The spatial positioning and distribution within organelles are fully dependent upon the morphological quality of tissue section on chips. Our data indicate that the combination of HE scores with RIN values to present both morphological integrity and rRNA completeness of tissues should be recommended as the important criteria before the performance of spatial transcriptomics and clarified in the method of spatial transcriptomic presentations. It would be more helpful if the quality of pathologists, number of red blood cells, detectability of tissue and cell structure, ratio of target cells within the area like malignant cells and density of cell nuclei can be considered in morphological digitalisation, scoring and evaluation.

In addition, more challenges of clinical sample preparations for comprehensive spatial transcriptome are to be faced and overcome. For example, it is a challenge to fill up or permeate with OCT into the alveolar space of resected human tissues, although the OCT embedding of ‘dry’ samples provided better morphological images. The OCT provides supportive effects within alveolar spaces and better quality of histological section, if OCT at the proper viscosity is perfused from the trachea into lungs at the certain pressure.^36^ It is hard to find the proper airway in the small pieces of human lung tissues removed from surgery for infusion of OCT, in order to avoid the transfigurations caused by the large number of cavities, fragmentation of ice crystals by high water content and wrong orientation of chip placement. We also found that the RNA degradation rate could be effectively reduced when the samples were temporarily preserved in drikold and transferred to –80℃. The compromised degree of sample RNA integrity was highly correlated with the repeating number of freeze‐thaw processes of samples. Another challenge is to select and define the ideal position, angle, integrity, shape, size and thickness of slices, in order to obtain optimal characteristics of spatial molecular images and positions. Finally, the annotation of the clustering is a challenge as well. There should not be so much astrocyte in lung tissue, as shown in Figure 6Q; the results may indicate that the annotation is beyond the annotated range of existing genes or that there are problems in gene expression in normal tissues.

With improvement of sample preparation, spatiotemporal positioning of gene and protein expression, intracellular metabolism and cell–cell communication can be visualised by spatiotemporal molecular omics.^1^, ^2^, ^37^, ^38^ The cell–cell communication controls the signalling and function between cells and maintains microenvironment homeostasis, including initiator cells, signal network factors, communicating conditions and receptor cells.^39^ Within tumour microenvironments, the cell–cell communication and interaction can control the microenvironmental ecology, regulate cancer cell growth and sensitivity to drugs and dominate the formation of inter‐cellular heterogeneity.^40^ The spatial cell–cell communication positions the interactions among cancer cells, immune cells, resident cells and endothelial cells; the activations of inflammatory mediators and the remodelling of localised metabolism. With deep understanding and rapid developments of clinical trans‐omics,^41^, ^42^, ^43^, ^44^ spatial trans‐omics of human samples demonstrates the exact distributions of decisive crossing points and nodes among various layers of multi‐omic molecular networks, the definite positioning of target pathways from gene and protein to function and regulation and the integration of clinical phenomes with spatial transcriptomic phenomes. Of those potentials and opportunities, the excellent quality of human sample preparation and handling will ensure the precise and reliable information generated from clinical spatial transcriptome.

In conclusion, our preliminary studies evaluated the potential challenges of human tissue preparation for clinical spatial transcriptome, in order to translate such technology into clinical practice. We categorise potential influencing factors from clinical aspects. Our data demonstrate that the combination of histological scores with RIN is critical to predict the quality of spatial transcriptomic samples. Clinical procedures of sample preparation should be furthermore optimised and standardised as new standards of operation performance for clinical spatial transcriptome, including patient selection, pathological definition, surgical types, sample harvest, temporary preservation conditions and solutions, frozen approaches, transport and storage conditions and duration. Our data suggested that the temporary preservation time and condition of samples at operation room should be within 30 min and in ‘dry’ status. The direct cryo‐preservation within OCT media for human lung sample is recommended. Thus, we believe that clinical spatial transcriptome will be a decisive approach and bridge in the development of spatiotemporal molecular images and provide new insights for understanding molecular mechanisms of diseases at multi‐orientations.

## CONFLICT OF INTEREST

The authors declare that they have no competing interests.

## CODE AVAILABILITY

Available at http://stereomap.cngb.org/ or https://202.108.211.75

## Supporting information

Supporting Information

Supporting Information

Supporting Information

Supporting Information

Supporting Information

## Data Availability

Fastq files of Stereo‐seq is available at http://stereomap.cngb.org/ and the corresponding metrics files is available Supplemental File 1. The high‐throughput sequencing data have been deposited into CNGB Sequence Archive of CNGBdb with accession number CNP0002374.
