# Supplementary material for: Clinical challenges of tissue preparation for spatial transcriptome
Source: Clin Transl Med. 2022 Jan 26;12(1):e669. doi: 10.1002/ctm2.669 (PMC8792118; doi:10.1002/ctm2.669)
Supplement: Supplementary file 2 — Supporting Information [file CTM2-12-e669-s002.docx]

| Table S1: HE and RIN score of lung samples. | | | | | | | | | | | | | |
| --- | --- | --- | --- | --- | --- | --- | --- | --- | --- | --- | --- | --- | --- |
| Sample Name | Tissue Location | Scoring items of HE | | | | | | | | | | **HE Score** | **RIN** |
|  |  | Tissue sectional integrity | Thickness uniformity | No knife marks or cracks | No wrinkles or folds | No pollutants | No bubbles | Good transparency | Nucleocytoplasmic contrast | No loose | Slice neatly and clearly numbered |  |  |
| Sample 1 | N | / | / | / | / | / | / | / | / | / | / | / | / |
|  | T | / | / | / | / | / | / | / | / | / | / | / | / |
| Sample 2 | N | / | / | / | / | / | / | / | / | / | / | / | / |
|  | T | / | / | / | / | / | / | / | / | / | / | / | / |
| Sample 3 | N | / | / | / | / | / | / | / | / | / | / | / | / |
|  | T | / | / | / | / | / | / | / | / | / | / | / | / |
| Sample 4 | N | / | / | / | / | / | / | / | / | / | / | / | / |
|  | T | / | / | / | / | / | / | / | / | / | / | / | / |
| Sample 5 | N | 10 | 10 | 10 | 10 | 10 | 10 | 6 | 5 | 10 | 10 | 91 | 2.8 |
|  | T | 10 | 10 | 10 | 10 | 10 | 10 | 10 | 10 | 10 | 10 | 100 | 5.5 |
| Sample 6 | N | 10 | 10 | 10 | 10 | 10 | 10 | 8 | 10 | 10 | 10 | 98 | 5.5 |
|  | T | 10 | 10 | 10 | 10 | 10 | 10 | 9 | 10 | 10 | 10 | 99 | 7.4 |
| Sample 7 | N | 10 | 10 | 10 | 10 | 10 | 7 | 10 | 5 | 10 | 10 | 92 | 5.4 |
|  | T | 10 | 10 | 10 | 10 | 10 | 10 | 3 | 5 | 10 | 10 | 88 | 4.9 |
| Sample 8 | N | 10 | 10 | 10 | 10 | 10 | 7 | 5 | 10 | 10 | 10 | 92 | 5 |
|  | T | 10 | 10 | 10 | 8 | 10 | 10 | 4 | 5 | 10 | 10 | 87 | 6.6 |
| Sample 9 | N | / | / | / | / | / | / | / | / | / | / | / | / |
|  | T | / | / | / | / | / | / | / | / | / | / | / | / |
| Sample 10 | N | 10 | 10 | 10 | 10 | 10 | 7 | 5 | 5 | 10 | 10 | 87 | 3.7 |
|  | T | 10 | 10 | 10 | 10 | 10 | 10 | 7 | 5 | 10 | 10 | 92 | 3.5 |
| Sample 11 | N | 10 | 10 | 10 | 10 | 10 | 10 | 7 | 5 | 10 | 10 | 92 | 4.6 |
|  | T | 10 | 10 | 10 | 10 | 10 | 10 | 9 | 10 | 10 | 10 | 99 | 7.1 |
| Sample 12 | N | 10 | 10 | 10 | 10 | 10 | 10 | 5 | 5 | 10 | 10 | 90 | 4.7 |
|  | T | 10 | 10 | 10 | 10 | 10 | 10 | 5 | 5 | 10 | 10 | 90 | 4.8 |
| Sample 13 | N | 10 | 10 | 10 | 10 | 10 | 7 | 3 | 5 | 10 | 10 | 85 | 5.8 |
|  | T | 10 | 10 | 10 | 10 | 10 | 7 | 5 | 5 | 10 | 10 | 87 | / |
| Sample 14 | N | 10 | 10 | 10 | 10 | 10 | 10 | 6 | 5 | 10 | 10 | 91 | 5.5 |
|  | T | 10 | 10 | 10 | 10 | 10 | 10 | 9 | 10 | 10 | 10 | 99 | 5.7 |
| Sample 15 | N | 10 | 10 | 10 | 10 | 10 | 10 | 3 | 0 | 10 | 10 | 83 | 4.6 |
|  | T | 10 | 10 | 10 | 8 | 10 | 10 | 6 | 5 | 10 | 10 | 89 | 4.7 |
| Sample 16 | N | 10 | 10 | 10 | 10 | 10 | 10 | 4 | 5 | 10 | 10 | 89 | 5.4 |
|  | T | 10 | 10 | 10 | 10 | 10 | 10 | 5 | 10 | 10 | 10 | 95 | 7 |
| Sample 17 | N | 10 | 10 | 10 | 8 | 10 | 10 | 7 | 5 | 10 | 10 | 90 | 5.1 |
|  | T | 10 | 10 | 10 | 10 | 10 | 10 | 7 | 5 | 10 | 10 | 92 | 3.9 |
| Sample 18 | N | 10 | 10 | 10 | 10 | 10 | 10 | 10 | 10 | 10 | 10 | 100 | 6 |
|  | P | 10 | 10 | 10 | 10 | 10 | 10 | 7 | 5 | 10 | 10 | 92 | 5.5 |
|  | T | 10 | 10 | 10 | 10 | 10 | 10 | 9 | 10 | 10 | 10 | 99 | 7.6 |
| Sample 19 | N | 10 | 10 | 10 | 10 | 10 | 10 | 10 | 10 | 10 | 10 | 100 | 6.5 |
|  | P | / | / | / | / | / | / | / | / | / | / | / | 2.6 |
|  | T | 10 | 10 | 10 | 10 | 10 | 10 | 10 | 10 | 10 | 10 | 100 | 6.8 |
| Sample 20 | N | 10 | 10 | 10 | 10 | 10 | 10 | 10 | 10 | 10 | 10 | 100 | 7.4 |
|  | P | 10 | 10 | 10 | 8 | 10 | 10 | 10 | 10 | 10 | 10 | 98 | 7.4 |
|  | T | 10 | 10 | 10 | 10 | 10 | 10 | 10 | 10 | 10 | 10 | 100 | 8.4 |
| Sample 20-2 | N | 10 | 10 | 10 | 10 | 10 | 10 | 10 | 5 | 10 | 10 | 95 | / |
|  | P | 10 | 10 | 10 | 10 | 10 | 10 | 10 | 5 | 10 | 10 | 95 | / |
|  | T | 10 | 10 | 10 | 10 | 10 | 10 | 10 | 10 | 10 | 10 | 100 | / |
| Sample 21 | N | 10 | 10 | 10 | 10 | 10 | 10 | 8 | 10 | 10 | 10 | 98 | 5.8 |
|  | P | 10 | 10 | 10 | 10 | 10 | 10 | 8 | 10 | 10 | 10 | 98 | 6.5 |
|  | T | 10 | 10 | 10 | 10 | 10 | 10 | 10 | 10 | 10 | 10 | 100 | 7.6 |
| Sample 22 | N | 10 | 10 | 10 | 10 | 10 | 10 | 3 | 0 | 10 | 10 | 83 | 5.2 |
|  | P | 10 | 10 | 10 | 10 | 10 | 10 | 4 | 0 | 10 | 10 | 84 | 4.9 |
|  | T | 10 | 10 | 10 | 10 | 10 | 10 | 3 | 5 | 10 | 10 | 88 | 6.4 |
| Sample 23 | N | 10 | 10 | 10 | 10 | 10 | 10 | 7 | 5 | 10 | 10 | 92 | 4.5 |
|  | P | 10 | 10 | 10 | 10 | 10 | 10 | 3 | 5 | 10 | 10 | 88 | 5 |
|  | T | 10 | 10 | 10 | 10 | 10 | 10 | 10 | 10 | 10 | 10 | 100 | 9.5 |
| Sample 24 | N | 10 | 10 | 10 | 10 | 10 | 10 | 6 | 5 | 10 | 10 | 91 | 5 |
|  | P | 10 | 10 | 10 | 10 | 10 | 10 | 5 | 5 | 10 | 10 | 90 | 4 |
|  | T | 10 | 10 | 10 | 10 | 10 | 10 | 4 | 5 | 10 | 10 | 89 | 7.2 |
| Sample 25 | N | 10 | 10 | 10 | 10 | 10 | 7 | 6 | 10 | 10 | 10 | 93 | 5.1 |
|  | T | 10 | 10 | 10 | 10 | 10 | 7 | 8 | 10 | 10 | 10 | 95 | 6.5 |
| Sample 26 | N | 10 | 10 | 10 | 10 | 10 | 10 | 3 | 10 | 10 | 10 | 93 | 6.9 |
|  | T | 10 | 10 | 10 | 10 | 10 | 10 | 9 | 10 | 10 | 10 | 99 | 7.3 |
| Sample 27 | N | 10 | 10 | 10 | 10 | 10 | 7 | 7 | 5 | 10 | 10 | 89 | 4 |
|  | P | 10 | 10 | 10 | 10 | 10 | 7 | 6 | 5 | 10 | 10 | 88 | 5.7 |
|  | T | 10 | 10 | 10 | 10 | 10 | 10 | 7 | 5 | 10 | 10 | 92 | 6.8 |
| Sample 28 | N | 10 | 10 | 10 | 10 | 10 | 10 | 7 | 10 | 10 | 10 | 97 | 7.4 |
|  | P | 10 | 10 | 10 | 10 | 10 | 10 | 7 | 10 | 10 | 10 | 97 | 7.4 |
|  | T | 10 | 10 | 10 | 8 | 10 | 10 | 10 | 10 | 10 | 10 | 98 | 8.2 |
| Sample 29 | N | 10 | 10 | 10 | 10 | 10 | 10 | 3 | 5 | 10 | 10 | 88 | 3.1 |
|  | T | 10 | 10 | 10 | 10 | 10 | 10 | 5 | 10 | 10 | 10 | 95 | 6.6 |
| Sample 30 | N | 10 | 10 | 10 | 8 | 10 | 10 | 9 | 10 | 10 | 10 | 97 | 5.7 |
|  | T | 10 | 10 | 10 | 10 | 10 | 10 | 8 | 10 | 10 | 10 | 98 | 6 |
| Sample 31 | N | 10 | 10 | 10 | 10 | 10 | 10 | 7 | 10 | 10 | 10 | 97 | 6.6 |
|  | P | 10 | 10 | 10 | 10 | 10 | 10 | 10 | 10 | 10 | 10 | 100 | / |
|  | T | 10 | 10 | 10 | 10 | 10 | 10 | 9 | 10 | 10 | 10 | 99 | 6.4 |
| Sample 32 | N | 10 | 10 | 10 | 10 | 10 | 10 | 5 | 10 | 10 | 10 | 95 | / |
|  | P | 10 | 10 | 10 | 10 | 10 | 10 | 10 | 5 | 10 | 10 | 95 | / |
|  | T | 10 | 10 | 10 | 10 | 10 | 10 | 10 | 10 | 10 | 10 | 100 | / |
| Sample 33 | N | 10 | 10 | 10 | 10 | 10 | 7 | 7 | 10 | 10 | 10 | 94 | 7.4 |
|  | T | 10 | 10 | 10 | 10 | 10 | 10 | 9 | 10 | 10 | 10 | 99 | 8.9 |
