# Supplementary material for: Clinical challenges of tissue preparation for spatial transcriptome
Source: Clin Transl Med. 2022 Jan 26;12(1):e669. doi: 10.1002/ctm2.669 (PMC8792118; doi:10.1002/ctm2.669)
Supplement: Supplementary file 3 — Supporting Information [file CTM2-12-e669-s001.docx]

| Table S2: The collected and processed information for each sample | | | | | | | |
| --- | --- | --- | --- | --- | --- | --- | --- |
| Sample Name | Tissue location | Sampling method | Preservation in operatin room | Transportation  and storage | Hours of sample during delivery | Sample processing method | Storage days at -80° |
| Sample 1 | N | tracheoscopy | dried, 4℃ | dried | 0.5h | LN+wet | 1 |
| Sample 1 | T | tracheoscopy | dried, 4℃ | dried | 0.5h | LN+wet | 1 |
| Sample 2 | N | tracheoscopy | dried, 4℃ | dried | 0.5h | LN+wet | 1 |
| Sample 2 | T | tracheoscopy | dried, 4℃ | dried | 0.5h | LN+wet | 1 |
| Sample 3 | N | tracheoscopy | dried, 4℃ | dried | 0.5h | LN+wet | 1 |
| Sample 3 | T | tracheoscopy | dried, 4℃ | dried | 0.5h | LN+wet | 1 |
| Sample 4 | N | surgery | saline，4℃ | saline | 2h | LN+wet | 14 |
| Sample 4 | T | surgery | saline，4℃ | saline | 2h | LN+wet | 14 |
| Sample 5 | N | surgery | saline，4℃ | saline | 2h | LN+wet | 10 |
| Sample 5 | T | surgery | saline，4℃ | saline | 2h | LN+wet | 10 |
| Sample 6 | N | surgery | saline，4℃ | saline | 2h | LN+wet | 6 |
| Sample 6 | T | surgery | saline，4℃ | saline | 2h | LN+wet | 6 |
| Sample 7 | N | surgery | saline，4℃ | saline | 2h | LN+wet | 3 |
| Sample 7 | T | surgery | saline，4℃ | saline | 2h | LN+wet | 3 |
| Sample 8 | N | surgery | dried, room temperature | saline | 1h | LN+wet | 2 |
| Sample 8 | T | surgery | dried, room temperature | saline | 1h | LN+wet | 2 |
| Sample 9 | N | surgery | saline，4℃ | saline | 2.5h | LN+wet | 2 |
| Sample 9 | T | surgery | saline，4℃ | saline | 2.5h | LN+wet | 2 |
| Sample 10 | N | surgery | saline，4℃ | saline | 2h | LN+wet | 1 |
| Sample 10 | T | surgery | saline，4℃ | saline | 2h | LN+wet | 1 |
| Sample 11 | N | surgery | saline，4℃ | saline | 2.5h | LN+wet | 15 |
| Sample 11 | T | surgery | saline，4℃ | saline | 2.5h | LN+wet | 15 |
| Sample 12 | N | surgery | dried, room temperature | saline | 1h | LN+wet | 15 |
| Sample 12 | T | surgery | dried, room temperature | saline | 1h | LN+wet | 15 |
| Sample 13 | N | surgery | saline，4℃ | saline | 2h | LN+wet | 12 |
| Sample 13 | T | surgery | saline，4℃ | saline | 2h | LN+wet | 12 |
| Sample 14 | N | surgery | saline，4℃ | saline | 2h | LN+wet | 10 |
| Sample 14 | T | surgery | saline，4℃ | saline | 2h | LN+wet | 10 |
| Sample 15 | N | surgery | dried, room temperature | saline | 1h | LN+wet | 9 |
| Sample 15 | T | surgery | dried, room temperature | saline | 1h | LN+wet | 9 |
| Sample 16 | N | surgery | dried, room temperature | saline | 1h | LN+wet | 8 |
| Sample 16 | T | surgery | dried, room temperature | saline | 1h | LN+wet | 8 |
| Sample 17 | N | surgery | saline，4℃ | saline | 2h | LN+wet | 5 |
| Sample 17 | T | surgery | saline，4℃ | saline | 2h | LN+wet | 5 |
| Sample 18 | N | surgery | dried, room temperature | saline | 1h | LN+wet | 11 |
| Sample 18 | P | surgery | dried, room temperature | saline | 1h | LN+wet | 11 |
| Sample 18 | T | surgery | dried, room temperature | saline | 1h | LN+wet | 11 |
| Sample 19 | N | surgery | saline，4℃ | dried | 1.5h | LN+Dry | 2 |
| Sample 19 | P | surgery | saline，4℃ | dried | 1.5h | LN+Dry | 2 |
| Sample 19 | T | surgery | saline，4℃ | dried | 1.5h | LN+Dry | 2 |
| Sample 20 | N | surgery | dried, room temperature | dried | 1h | LN+Dry | 2 |
| Sample 20 | P | surgery | dried, room temperature | dried | 1h | LN+Dry | 2 |
| Sample 20 | T | surgery | dried, room temperature | dried | 1h | LN+Dry | 2 |
| Sample 20-2 | N | surgery | dried, room temperature | dried | 1h | LN+wet | 2 |
| Sample 20-2 | P | surgery | dried, room temperature | dried | 1h | LN+wet | 2 |
| Sample 20-2 | T | surgery | dried, room temperature | dried | 1h | LN+wet | 2 |
| Sample 21 | N | surgery | dried, room temperature | dried | 1h | OCT+ Isopentane+LN+Dry | 1 |
| Sample 21 | P | surgery | dried, room temperature | dried | 1h | OCT+ Isopentane+LN+Dry | 1 |
| Sample 21 | T | surgery | dried, room temperature | dried | 1h | OCT+ Isopentane+LN+Dry | 1 |
| Sample 22 | N | surgery | saline，4℃ | saline | 1.5h | LN+wet | 37 |
| Sample 22 | P | surgery | saline，4℃ | saline | 1.5h | LN+wet | 37 |
| Sample 22 | T | surgery | saline，4℃ | saline | 1.5h | LN+wet | 37 |
| Sample 23 | N | surgery | dried, room temperature | saline | 1h | LN+wet | 37 |
| Sample 23 | P | surgery | dried, room temperature | saline | 1h | LN+wet | 37 |
| Sample 23 | T | surgery | dried, room temperature | saline | 1h | LN+wet | 37 |
| Sample 24 | N | surgery | dried, room temperature | saline | 1h | LN+wet | 36 |
| Sample 24 | P | surgery | dried, room temperature | saline | 1h | LN+wet | 36 |
| Sample 24 | T | surgery | dried, room temperature | saline | 1h | LN+wet | 36 |
| Sample 25 | N | surgery | saline，4℃ | saline | 2h | LN+wet | 35 |
| Sample 25 | T | surgery | saline，4℃ | saline | 2h | LN+wet | 35 |
| Sample 26 | N | surgery | saline，4℃ | saline | 2h | LN+wet | 35 |
| Sample 26 | T | surgery | saline，4℃ | saline | 2h | LN+wet | 35 |
| Sample 27 | N | surgery | dried, room temperature | saline | 1h | LN+wet | 30 |
| Sample 27 | P | surgery | dried, room temperature | saline | 1h | LN+wet | 30 |
| Sample 27 | T | surgery | dried, room temperature | saline | 1h | LN+wet | 30 |
| Sample 28 | N | surgery | saline，4℃ | saline | 2.5h | LN+wet | 30 |
| Sample 28 | P | surgery | saline，4℃ | saline | 2.5h | LN+wet | 30 |
| Sample 28 | T | surgery | saline，4℃ | saline | 2.5h | LN+wet | 30 |
| Sample 29 | N | surgery | saline，4℃ | saline | 2h | LN+wet | 27 |
| Sample 29 | T | surgery | saline，4℃ | saline | 2h | LN+wet | 27 |
| Sample 30 | N | surgery | dried, 4℃ | dried | 2h | OCT+ Isopentane+LN+Dry | 7 |
| Sample 30 | T | surgery | dried, 4℃ | dried | 2h | OCT+ Isopentane+LN+Dry | 7 |
| Sample 31 | N | surgery | dried, room temperature | dried | 0.5h | OCT+drikold+Dry | 1 |
| Sample 31 | P | surgery | dried, room temperature | dried | 0.5h | OCT+drikold+Dry | 1 |
| Sample 31 | T | surgery | dried, room temperature | dried | 0.5h | OCT+drikold+Dry | 1 |
| Sample 32 | N | surgery | dried, room temperature | dried | 0.5h | OCT+drikold+Dry | 4h |
| Sample 32 | P | surgery | dried, room temperature | dried | 0.5h | OCT+drikold+Dry | 4h |
| Sample 32 | T | surgery | dried, room temperature | dried | 0.5h | OCT+drikold+Dry | 4h |
| Sample 33 | N | surgery | saline，4℃ | saline | 2h | OCT+drikold+wet | 1 |
| Sample 33 | T | surgery | saline，4℃ | saline | 2h | OCT+drikold+wet | 1 |
