# Supplementary material for: Clinical challenges of tissue preparation for spatial transcriptome
Source: Clin Transl Med. 2022 Jan 26;12(1):e669. doi: 10.1002/ctm2.669 (PMC8792118; doi:10.1002/ctm2.669)
Supplement: Supplementary file 4 — Supporting Information [file CTM2-12-e669-s004.docx]

| Table S3: The quality of Stereo-Seq data | | | | |
| --- | --- | --- | --- | --- |
| Sample Name | mean UMI number (bin50) | Saturability | Sequencing Total Reads/M | mean gene number (bin50) |
| sample2N | 584.6927281 | 30.39% | 123.77/2 | 495.7654468 |
| sample2T | 757.8618726 | 30.39% | 123.77/2 | 639.1836953 |
| sample9N | 47.47133459 | 42.44% | 260.45 | 30.8381109 |
| sample9T | 153.5411255 | 36.69% | 246.27 | 98.47619048 |
| sample10N (Figure 6 A, E, I and M) | 104.6048685 | 35.06% | 211.58 | 70.93850946 |
| sample10T (Figure 6 B, F, J and N) | 70.342855 | 39.89% | 209.39 | 46.74802676 |
| sample19P | 4.757484023 | 3.94% | 150.86 | 4.353010427 |
| sample19T | 369.7436145 | 16.73% | 256.36 | 302.5318238 |
| sample21N | 468.5369554 | 19.97% | 192.72 | 349.3579114 |
| sample21P | 151.9319102 | 7.39% | 70.42 | 121.6627258 |
| sample21T | 226.7536087 | 9.54% | 111.95 | 181.228598 |
| sample30N | 6880.90872 | 61.36% | 3.41G | 2741.254246 |
| sample30T | 733.3303383 | 15.81% | 2.38G/2 | 468.2756636 |
| sample31N | 807.0272509 | 11.39% | 104.51 | 581.4189153 |
| sample31P | 2112.621273 | 84.54% | 3.20G | 803.7560124 |
| sample31T | 200.9547078 | 5.65% | 96.51/2 | 165.1866191 |
| sample32N | 355.4549112 | 53.72% | 210.49 | 233.9915612 |
| sample32P | 643.6462672 | 27.65% | 214.8 | 395.7135572 |
| sample32T | 358.4624518 | 54.38% | 178.48 | 228.9323241 |
| sample33N (Figure 6 C, G, K and O) | 1368.983909 | 87.60% | 3.26G | 591.6133427 |
| sample33T (Figure 6 D, H, Land P) | 4709.556464 | 73.97% | 2.94G | 1643.274664 |
