# Supplementary material for: Clinical challenges of tissue preparation for spatial transcriptome
Source: Clin Transl Med. 2022 Jan 26;12(1):e669. doi: 10.1002/ctm2.669 (PMC8792118; doi:10.1002/ctm2.669)
Supplement: Supplementary file 5 — Supporting Information [file CTM2-12-e669-s003.docx]

| Table S4: Rules for HE images score | |
| --- | --- |
| Quality standard | Deduction items |
| Tissue slice integrity. | Slightly incomplete tissue slice: minus 1-3 points; incomplete tissue slice: minus 4-10 points. |
| The thickness of slice is 3-5μm and uniform. | Tissue overlaps resulted in diagnosis problems, minus 6-10 points; Uneven thickness, minus 3-5 points. |
| Slice without knife marks. | Tissue slice with knife marks and cracks not affect the diagnosis, minus 2 points; Imaging diagnosis, minus 5 points. |
| The slice is flat without folds. | Folds not affect the diagnosis, minus 2 points; Folds resulted in diagnosis problems, minus 5 points each. |
| No contaminants. | With contaminants, minus 10 points. |
| No bubbles, no neutral balsam spill. | With bubbles, minus 3 points; neutral balsam spill, minus 3 points. |
| Good transparency. | Poor transparency, minus 1-3 points; fuzzy tissue structure, minus 3-7 points. |
| The nuclei and cytoplasm were clearly stained. | The nucleus is grayish or too blue, minus 5 points; unclear contrast between red (cytoplasm) and blue (nucleus), minus 5 points. |
| The slices are not loose and the tissue position is appropriate. | Loose slice, minus 5 points; slice improper position, minus 5 points. |
| The slice is neat, the label is straight and firm, and the number shall be clear. | Untidy slices or unfirmly labels, minus 3 points each; unclear number, minus 4 points. |
